# miR-449, identified through antiandrogen exposure, mitigates functional biomarkers associated with ovarian cancer risk

**DOI:** 10.1038/s41598-024-80173-z

**Published:** 2024-12-02

**Authors:** Xia Wang, Ho-Hyung Woo, Michelle Wei, Steven Gibson, Mitzi Miranda, Demaretta Rush, Janiel Cragun, Wenxin Zheng, Guang Yao, Setsuko K. Chambers

**Affiliations:** 1 College of Animal Science and Technology, Northwest A&F University, Shaanxi, China; 2 Department of Molecular and Cellular Biology, University of Arizona, Tucson, AZ USA; 3 Department of Obstetrics and Gynecology, University of Arizona, Tucson, AZ USA; 4 Department of Pathology, University of Arizona, Tucson, AZ USA; 5 Department of Pathology, University of Texas Southwestern Medical Center, Dallas, TX USA; 6 University of Arizona Cancer Center, Tucson, AZ USA

**Keywords:** MicroRNA, CSF1R, Androgen receptor, Flutamide, Ovarian cancer risk, Computational biology and bioinformatics, Biomarkers, Oncology

## Abstract

The involvement of the androgen receptor (AR) pathway in developing epithelial ovarian cancer is increasingly acknowledged. However, the specific mechanisms by which anti-androgen agents, such as flutamide, may prevent ovarian cancer and their efficacy remain unknown. This study was initiated by investigating the impact of flutamide on miRNA expression in women at high risk (HR) for ovarian cancer. Ovarian and tubal tissues, free from ovarian, tubal, peritoneal cancers, and serous tubal intraepithelial carcinoma (STIC), were collected from untreated and flutamide-treated HR women as well as low-risk (LR) women controls. We performed miRNA sequencing on these 3 sample cohorts and observed that flutamide normalized miRNA levels in HR tissues, notably upregulating the miR-449 family to levels seen in LR tissues. In subsequent tests in primary ovarian epithelial cells and ovarian cancer cell lines (SKOV3 and Hey), flutamide also increased miR-449a and miR-449b-5p levels. Introducing mimics of these miRNAs reduced the mRNA and protein levels of AR and colony-stimulating factor 1 receptor (CSF1R, also known as *c-fms*), both of which are known contributors to ovarian cancer progression, with emerging evidence also supporting their roles in ovarian cancer initiation. Ovarian cancer cell migration was inhibited upon introducing miR-449a and miR-449b-5p mimics. Together, our study suggests a novel dual-inhibitory mechanism of flutamide on the AR pathway (AR expression suppression in addition to direct androgen antagonism) and supports its chemopreventive potential in ovarian cancer, especially for HR patients with low miR-449 expression.

**Supplementary Information:**

The online version contains supplementary material available at 10.1038/s41598-024-80173-z.

## Introduction

Post-transcriptional RNA regulation, including the crucial role of microRNAs (miRNAs), is pivotal in modulating gene expression with profound impacts on various cellular activities. Translation of our basic post-transcriptional RNA research^1–4 ^to the pursuit of risk stratification and prevention potential for women who are at HR for developing ovarian cancer^5–8^ underlies the foundation of this report.

This study was informed by our previous in vitro and in vivo findings that underscored the potential role of androgen signaling and AR pathway in epithelial ovarian cancer development^9^, which steered us towards evaluating anti-androgens, such as flutamide, as a potential preventive measure for HR women. Furthering this line of inquiry, we also conducted a parallel study to assess biomarkers of ovarian cancer risk in HR versus LR women^10^. In the current report, we used prospectively collected biospecimens in three predefined cohorts (LR, HR, and flutamide-treated HR) from a phase 2 study (flutamide IIT NCT00699907) to delineate global variations in miRNA profiles across these cohorts, assess the role of flutamide in modulating miRNA expression, and test the effects of modulated miRNAs on potential biomarkers in HR women.

In our previous targeted immunohistochemical analysis of ovarian and fallopian tube tissues from these clinical studies, overexpression of CSF1/CSF1R (macrophage colony-stimulating factor and its receptor) and ErbB4 proteins in tissues from HR women were observed compared to LR women. Notably, this overexpression was attenuated in HR women who underwent flutamide treatment. In fact, CSF1 and ErbB4 expression levels were found to have high predictive value for flutamide sensitivity in ovarian tissues^9^. Together, our findings underscore the potential involvement of cellular pathways such as CSF1/CSF1R and AR in the development of epithelial ovarian cancer and the response to flutamide treatment^9,10^.

Considering the extensive roles of miRNAs in governing RNA regulation and subsequent cellular processes, we hypothesize that miRNA profiling may differentiate HR and LR cohorts. We also postulate that flutamide treatment may, to some extent, correct the aberrant miRNA profile observed in HR individuals, potentially impacting functional biomarkers such as CSF1/CSF1R.

Specifically, we aim to identify miRNAs differentially expressed between HR and LR women which regulate the dysregulated CSF1/CSF1R and AR pathways in HR patients. For this, we analyzed tissue samples from HR women, comparing them to both flutamide-treated HR and LR samples. Notably, our study excluded women diagnosed with ovarian or other peritoneal cancers. This exclusivity sets our research apart, as we focus solely on morphologically and pathologically normal ovary and fallopian tube samples from cancer-free women. In contrast, existing literature primarily examines miRNA profiles in women already diagnosed with ovarian cancer^11–15^. We are not aware of previous reports outside of our own, which focus on miRNA profiling in normal tissues of women at HR for ovarian cancer.

In this study, our miRNA-sequencing results highlighted that flutamide treatment leads to a highly significant upregulation of the miR-449 cluster in the HR cohort, effectively restoring its expression to levels seen in LR women. Subsequent in vitro analyses in ovarian cells corroborated that miR-449a and miR-449b-5p reduce the expression of CSF1R and AR. Consistent with CSF1R and AR pathways being known contributors to ovarian cancer progression and initiation and being significantly elevated in HR women, we observed that miR-449a and miR-449b-5p reduced the migration of ovarian cancer cells. Remarkably, our study unveiled a novel mechanism wherein the anti-androgen flutamide suppresses AR expression by upregulating miR-449, complementing its well-known role as a direct-binding AR inhibitor. Collectively, these data both validate our miRNA profiling approach and strengthen the basis for flutamide as a chemopreventive approach in HR women with downregulated miR-449 expression.

## Methods

*Definition of high-risk (HR). *HR women were identified based on the following criteria in this study: carrying a BRCA1 or BRCA2 mutation, a Lynch Syndrome mutation, and/or having a family history characterized by one or more first-degree relatives with epithelial ovarian cancer, one or more cases of breast cancer diagnosed at age 40 or younger, more than one case of breast cancer diagnosed at age 50 or younger, instances of male breast cancer, and/or a family history of breast/ovarian cancer. Among the HR group, there was a higher prevalence of BRCA2 mutations compared to BRCA1, representative of our HR clinic population^6^. Low-risk (LR) patients did not meet any of these HR criteria. A personal history of breast cancer was present in both HR and LR populations^9,10^.

*Clinical matching of patient cohorts. *This report is a validation of a notable finding from our initial miRNA sequencing data obtained in tissues from the Flutamide and its parallel clinical trials^9,10^. We designed a comparison across 3 cohorts (LR, HR, and flutamide-treated HR). The flutamide-treated HR cohort consisted of 12 available patients, and for a balanced comparison, similar numbers of patients from the HR (*n* = 12) and LR (*n* = 13) groups were selected, considering matching clinical parameters and tissue sample collection.

The HR and LR cohorts were chosen to match as closely as possible to the flutamide treated-HR cohort and to each other in terms of menopausal status (post-menopausal vs. peri- or pre-menopausal) and Body Mass Index (BMI). There were 9 of 12 (75%) peri-/pre-menopausal patients in the HR cohort and 9 of 13 (69%) such patients in the LR cohort, compared to 9 of 12 (75%) in the flutamide-treated HR cohort. The median BMI (range) in the LR cohort was 27.9 kg/m^2^ (19.9–36.0), compared to 27.1 kg/m^2^ (21.0–36.9) in the HR cohort and 25.5 kg/m^2^ (19.3–45.0) in the flutamide-treated HR cohort. Additionally, the HR and flutamide-treated HR cohorts were matched for BRCA1/2 status, with 9 of 12 (75%) patients in each cohort carrying either a BRCA1 or BRCA2 germline mutation.

*Sample preparation and small RNA extraction.* Deidentified fixed fallopian tube and ovary tissues were obtained following patient consent, as outlined in the section on clinical matching of patient cohorts. Both ovarian and fallopian tube samples were included in this study, considering that the fallopian tubal epithelium has been accepted as one origin of ovarian epithelial carcinoma. Importantly, all tissue samples were confirmed to be free from ovarian, tubal, peritoneal cancers, tubal dysplasia, and serous tubal intraepithelial carcinoma (STIC), as determined by our Gynecologic Oncology pathologists. Tissue sections (10 μm) were prepared from each ovarian and tubal sample. In instances where ovary and tube samples were found embedded within the same block, they were meticulously separated and re-embedded. We note that ovarian tissue blocks from 2 of 13 LR cases were unable to be retrieved from archives, with 2 of 12 fallopian tube blocks unavailable from the flutamide-treated HR cases.

Small RNAs were extracted from an average of 10 sections per sample using the miRNeasy FFPE kit (Qiagen). Quality tests, including qRT-PCR and gel electrophoresis, were used to confirm successful miRNA isolation without contamination. Our miRNA analysis included a total of 12 HR, 12 flutamide-treated HR, and 11 LR ovarian samples, along with a total of 12 HR, 10 flutamide-treated HR, and 13 LR fallopian tube samples.

*miRNA-sequencing and analysis.* Small RNA libraries were constructed and sequenced following a standardized procedure at GENEWIZ (South Plainfield, NJ). Libraries were prepared using the TruSeq Small RNA library Prep Kit (Illumina, San Diego, CA), validated on the Agilent TapeStation 4200 (Agilent Technologies, Palo Alto, CA, USA), and quantified using a Qubit 2.0 Fluorometer (Invitrogen, Carlsbad, CA). Sequencing was performed on an Illumina HiSeq instrument in a 2 × 150 bp paired-end configuration. The sequencing reads have been deposited in the Gene Expression Omnibus (GEO) database (GSE252170).

For data processing, sequencing adapters and low-quality bases were trimmed using Cutadapt^16^. Reads with 18–26 nt in length were kept, and the paired-end reads were merged using fastq-join^17^. Reads mapping and miRNA quantification were performed using miRMaster with the default parameter settings^18,19^. Differential expression analysis was performed using DESeq2^20^ with a negative binomial generalized linear model. A miRNA was considered differentially expressed between two sample groups if it exhibited > 2-fold difference in mean expression with a p-value < 0.05. Potential mRNA targets of miRNAs were identified using the miRDB database^21^, based on MirTarget predictions derived from extensive miRNA-target interaction analyses^22^.

*Cell culture and flutamide treatment.* SKOV3 and Hey human ovarian cancer cells were cultured in Dulbecco’s Modified Eagle/F12 Ham’s medium (DMEM/F12) with 10% NuSerum (Corning). Primary ovarian epithelial cells (Cell Biologics, Chicago, IL) were cultured in DMEM/F12 with hEGF (400 pg/ml) and 10% NuSerum. The HR or LR origin of these primary ovarian epithelial cells is not known. The limited information (donor age between 56 and 60 years) may indicate a LR rather than HR origin, as HR women would typically undergo removal of normal ovaries at an earlier age. All cells were incubated at 37 °C with 5% CO_2_. For flutamide treatment, cells were cultured in DMEM/F12 supplemented with 1% NuSerum for 24 h, followed by a 24-hour treatment with flutamide (5 µM) in the same medium before being harvested for miRNA expression analysis.

*Quantitative reverse transcription PCR (qRT-PCR).* Total RNA was extracted with Trizol (Invitrogen) and treated with RNase-free DNase (Promega, USA). For miRNA quantification, stem-loop qRT-PCR was used to enhance amplification specificity^23^. Following cDNA synthesis of miRNAs and 5 S rRNA internal control, quantitative PCR (qPCR) was conducted using an Eppendorf Realplex2 instrument. The thermal cycling conditions included a 10-minute initial denaturation at 95 °C, followed by 40 cycles of 15 s at 95 °C and 1 min at 60 °C. Final miRNA expression values were calculated using the ∆∆Ct method^24^. Similarly, for mRNA quantification, CSF1R and AR transcripts were reverse transcribed by pdN6 random hexamers and quantified with qPCR, with GAPDH transcript used as an internal control. All qRT-PCR primer sequences are detailed in Table 1.

**Table 1:** Primers used for qRT-PCR.

For reverse transcription	
miR-449a	gtcgtatccagtgcagggtccgaggtattcgcactggatacgacaCCaGCT
miR-449b-5p	gtcgtatccagtgcagggtccgaggtattcgcactggatacgacGCCaGCT
miR-449c-5p	gtcgtatccagtgcagggtccgaggtattcgcactggatacgacaCaGCCG
Human 5 S rRNA	gtcgtatccagtgcagggtccgaggtattcgcactggatacgacCAGGCG
**For miRNA qPCR**	
miR-449a-forward	ggctgtGGCAGtGtAttGtt
miR-449b-5p-forward	ggctgAGGCAGtGtAttGtt
miR-449c-5p-forward	ggctgtAGGCAGtGtAttGCtAG
Human 5 S rRNA-forward	CTGGTTAGTACTTGGACGGGAGAC
Universal reverse primer	gtgcagggtccgaggt
**For mRNA qPCR**	
CSF1R-forward	CCAGGCTCTTTGGGGCTAGACAGACTGG
CSF1R-reverse	GGGCCTGAGCTGAGTGTGGTCTGTGAGC
AR-forward	TGACCCACAGGTCCTGTGAAGGAGC
AR-reverse	TCTTCCAACAGCTCAGGTCCCATTAGG
GAPDH-forward	AACAGCGACACCCACTCCTCCACC
GAPDH-reverse	CCAGCAGTGAGGGTCTCTCTCTTCCTC

*Transient transfection with miRNA mimics.* Cells were plated on 6-well plates one day prior to transfection. Mimics of miR-449a and miR-449b-5p were purchased from Sigma (MISSION microRNA Mimics). Transfection of miRNA mimics was performed according to the manufacturer’s instructions, alongside MISSION miRNA negative control. For subsequent mRNA level measurement of miRNA target genes using qRT-PCR, transfection was done in 12-well plates with 10 femtomoles (fmol) miRNA mimics or negative control in each well. For subsequent protein level measurement of miRNA target genes using Western, transfection was done in T25 flasks with 60 fmol miRNA mimics or negative control in each flask.

*Immunoblotting.* Cytoplasmic lysates (140 µg), isolated with NE-Per (Thermofisher, USA), were subjected to SDS-PAGE and electroblotted onto a PVDF membrane. The membranes were incubated with mouse monoclonal anti-c-fms (sc-46662, Santa Cruz Biotech, USA), anti-AR (sc-7305, Santa Cruz Biotech, USA), and anti-Pan Actin (ACTN05, NeoMarkers, Fremont, CA) antibodies. Following washes with PBS-T (PBS with 0.1% Tween-20) and incubation with anti-mouse (HRP)-conjugated secondary antibody, proteins were detected using SuperSignal chemiluminescence (Pierce). The large amount (140 µg) of total protein loaded was mainly to ensure adequate detection of CSF1R, which is typically expressed at low levels in the cells tested. To prevent oversaturation of the actin control bands, we used a significantly shorter exposure time for the actin blot (15 s) than the CSF1R blot (20 min).

*Directed migration assay.* Cell migration was quantified using Membrane Migration Culture Systems (Sigma, USA) as previously described^2^. Briefly, fibronectin (25 µg/ml, Fisher) was added as a chemoattractant in the lower wells of a 24-well plate, above which uncoated filters (8-µm pore) were placed. Cells (5 × 10^4^ per well) were seeded onto the uncoated filters and incubated for 20 h. Subsequently, the top chamber cells were wiped off with Q-tips and the top and bottom chambers were washed with cold PBS, then dried and frozen at −80 °C for 30 min. Bottom chamber cells were quantified by the lysis method using CyQuant Cell Proliferation Assay Kit (Invitrogen) as per the manufacturer’s protocol, with the obtained fluorescence intensity (relative fluorescence units, RFU) directly correlated with the number of migrated cells.

*Statistical analysis.* Unless otherwise noted, data are presented as mean ± s.e.m. derived from experiments conducted in triplicate, analyzed using R with a one-sided unpaired t-test, considering a p-value < 0.05 indicative of statistical significance.

## Results

*miR-449 downregulation is associated with a high risk for ovarian cancer and can be reverted by flutamide treatment*.

In this study, we first conducted miRNA-sequencing profiling in the ovary and fallopian tube, from HR and LR patients (high- and low-risk for ovarian cancer, respectively). HR group was further divided into two cohorts: those treated with flutamide (the “flutamide” cohort) and those not treated with flutamide (the “HR” cohort). The samples across the three cohorts (flutamide, HR, and LR) were matched for body mass index (BMI) and menopausal status. The HR and flutamide cohorts were defined for the high-risk status and balanced for germline BRCA1/2 conditions, as detailed in Methods.

Our analysis revealed notable differences in miRNA expression profiles between these cohorts. When comparing the flutamide cohort to the HR cohort, there was a significantly greater number of significantly up-regulated miRNAs than down-regulated (Suppl. Figure 1 A). A similar, though not as statistically significant, pattern was observed when comparing the LR cohort to the HR cohort (Suppl. Figure 1B). These findings suggest a general trend where the HR status is associated with a lower miRNA expression profile compared to LR in both the ovary and tube. Interestingly, this pattern appears to be reversed with flutamide treatment, indicating a potential regulatory role of flutamide in miRNA expression in these tissues.

**Fig. 1:**
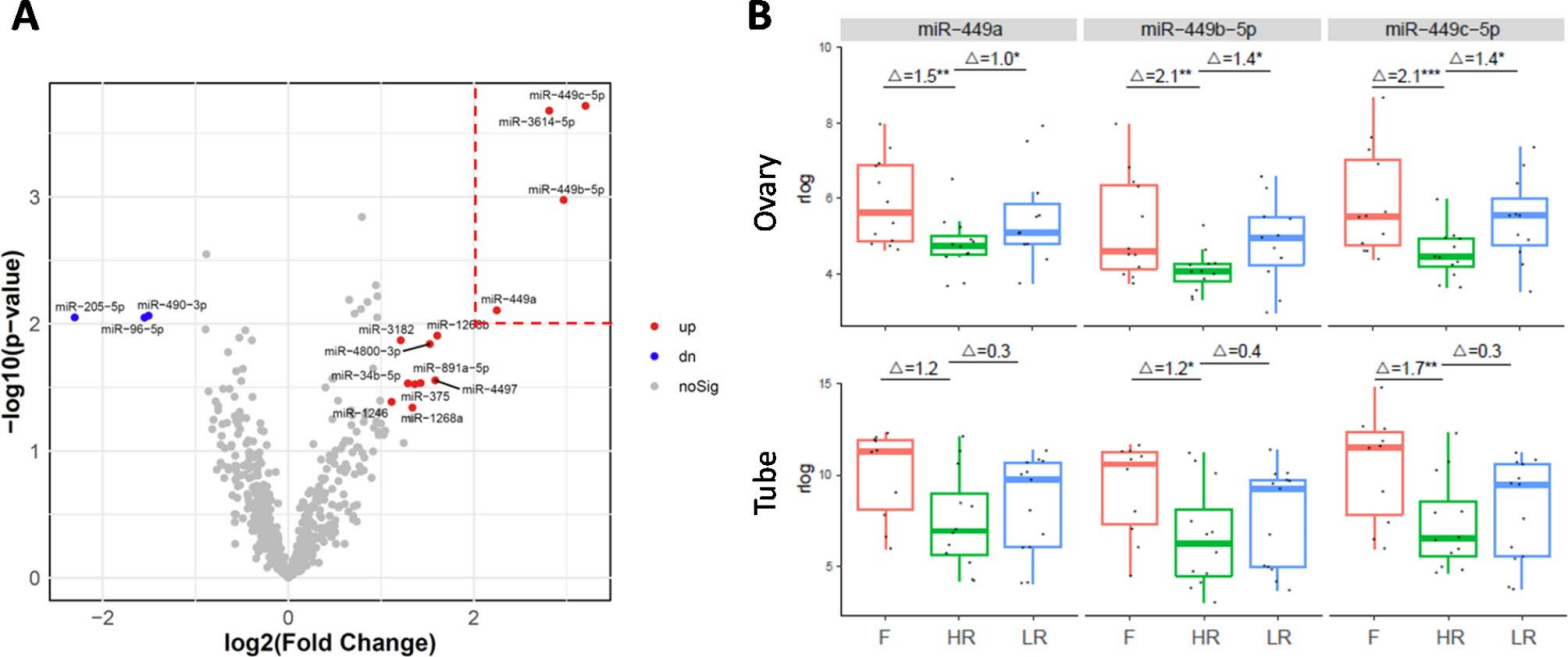
The miR-449 family is strongly associated with ovarian cancer HR and flutamide action. (**A**) Volcano plot of miRNA expression profile in flutamide vs. HR cohort from the ovary samples. Red and blue dots indicate miRNAs that are significantly up-regulated (fold change > 2, *p* < 0.05) and down-regulated (fold change < 0.5, *p* < 0.05), respectively. (**B**) Boxplot of the expression (in rlog normalized values, y-axis) of miR-449 family (miR-449a, left; miR-449b-5p, middle; miR-449c-5p, right) in the cohorts of flutamide (F), HR, and LR in the ovary (top) and fallopian tube (bottom). The effect size (Glass’s delta) and statistical significance (*, **, *** indicating *p* < 0.05, 0.01, 0.001, respectively) for each pairwise comparison based on DESeq2 differential analysis are shown at the top of each boxplot.

Among these miRNA profiles (Suppl. Figure 1), the most striking difference emerged from comparing the flutamide and HR cohorts in the ovary. Specifically, miR-449a, miR-449b-5p, and miR-449c-5p from the miR-449 family were identified among the most significantly up-regulated miRNAs (top right corner beyond the two red-dashed threshold lines, indicating fold change > 4 and *p* < 0.01, respectively; Fig. 1A). A similar trend of significant upregulation in miR-449a, miR-449b-5p, and miR-449c-5p was observed when comparing ovarian LR and HR cohorts (Suppl. Figure 1B, left). That is, these miRNAs were highly expressed in LR, reduced in HR, and increased again with flutamide treatment in the ovary, a pattern confirmed by large effect size (Glass’s delta > 0.8) and statistical significance (*p* < 0.05; Fig. 1B, top). Interestingly, this expression pattern of the three miR-449 family members was also mirrored, albeit with a smaller effect size and statistical significance, in fallopian tube samples that were matched to the ovarian samples in our study (Fig. 1B, bottom).

As an independent validation of the miRNA-seq results, we conducted qRT-PCR analysis on miR-449 expression using the original tissue samples. The qRT-PCR results corroborated the miRNA-seq data, showing not only a strong and significant correlation in miR-449 expression measurements across individual samples (Suppl. Figure 2 A) but also a consistent differential expression pattern between patient cohorts (high in LR, low in HR, and restored to high again following flutamide treatment) with even larger effect sizes than those observed in miRNA-seq (Suppl. Figure 2B). These consistent observations, across both miRNA-seq and qRT-PCR analyses, suggest that in ovarian cancer-related tissues, the reduced expression of miR-449 family members correlates with the HR status for ovarian cancer, and flutamide treatment restores the expression of these miRNAs. Such miR-449-related patterns were particularly evident in the ovary, and thus the tissue focus of our subsequent analysis.

*miR-449a and miR-449b-5p suppression of CSF1R and AR expression as a link to flutamide action and ovarian cancer risk reduction*.

**Fig. 2:**
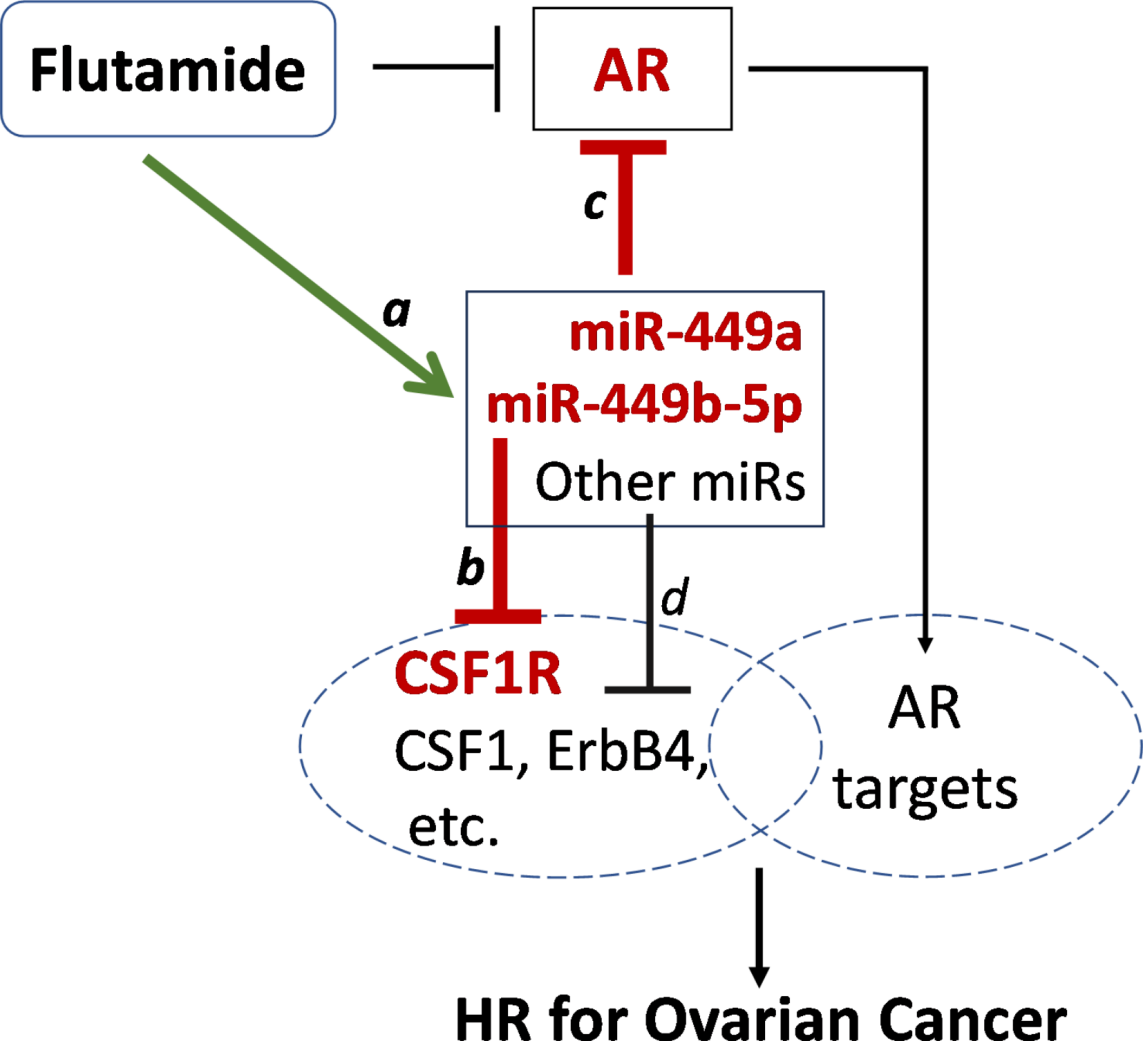
Proposed working model of flutamide action on miR-449 expression and downstream targets. a, Flutamide up-regulates miR-449a and miR-449b-5p (and other miRNAs); b, miR-449a and miR-449b-5p suppress CSF1R expression; c, miR-449a and miR-449b-5p suppress AR expression. d, Other flutamide-upregulated miRNAs may suppress CSF1, ErbB4, etc.

Next, we investigated whether and how miR-449a, miR-449b-5p, and miR-449c-5p may contribute mechanistically to the HR vs. LR status and flutamide action. Previously, we observed that (i) androgen signaling promotes ovarian cancer development^9^, and (ii) CSF1/CSF1R and ErbB4 proteins were overexpressed in the ovarian tissues of HR women compared to LR, and this overexpression was reversed in those HR women treated with flutamide^9,10^. Following these clues, we explored the computationally predicted mRNA targets of miRNAs associated with these HR-related proteins, based on the MirTarget tool in the miRDB database^21^. This analysis revealed that miR-449a and miR-449b-5p, but not miR-449c-5p, potentially target CSF1R and AR mRNAs (Suppl. Figure 3). Based on these findings, we proposed a potential working model of flutamide action as shown in Fig. 2.

Our proposed working model posits the following sequence of potential events: first, given that miR-449a and miR-449b-5p may target and repress CSF1R expression (link *b*, Fig. 2; Suppl. Figure 3), the downregulation of miR-449a and miR-449b-5p in HR relative to LR (Fig. 1B) could lead to the observed CSF1R overexpression in HR women^5^. Second, following flutamide treatment, miR-449a and miR-449b-5p are upregulated (link *a*, Fig. 2; Fig. 1), which could reverse the overexpressed CSF1R in HR women, consistent with the findings in^5^. Third, the computationally predicted inhibitory effects of miR-449a and miR-449b-5p on AR expression (link *c*, Fig. 2; Suppl. Figure 3), if experimentally validated, could reveal a novel mechanistic link of flutamide action – that is, in addition to its established role as an AR antagonist by blocking the binding of androgens to AR, flutamide may also indirectly suppress AR expression by upregulating miR-449a and miR-449b-5p. In addition, we noticed that flutamide significantly upregulates several other miRNAs in HR ovarian and fallopian tube tissues (Suppl. Figure 1 A), which could target and suppress other HR markers such as CSF1, ErbB4, and possibly some AR targets (link *d*, Fig. 2). Our subsequent experiments, guided by this proposed working model, primarily focuses on validating the upregulation of miR-449a and miR-449b-5p by flutamide in the ovary and testing their consequent effects on CSF1R and AR expression (links *a-c*, Fig. 2). Notably, connecting links *a* and *b*would provide a mechanistic explanation for our previously observed phenomenon of down-regulation of CSF1R by flutamide in HR women^9^.

*Flutamide upregulates miR-449a and miR-449b-5p expression in both primary epithelial and malignant ovarian cells*

**Fig. 3:**
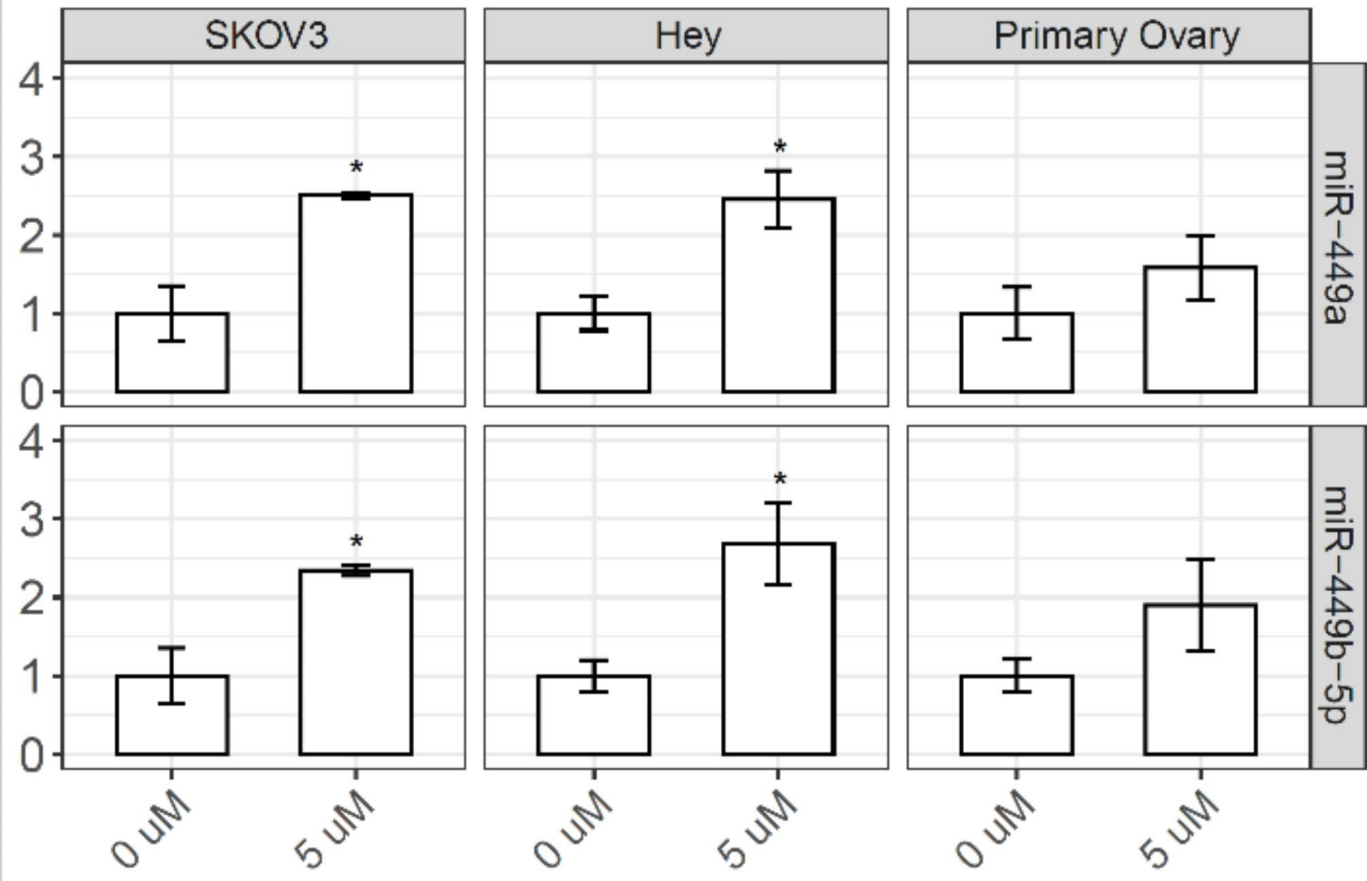
Flutamide up-regulates miR-449a and miR-449b-5p in both primary epithelial and malignant ovarian cells. Cells were treated with flutamide (5 µM) or vehicle control (0 µM) for 24 h, and the expression of miR-449a (top) and miR-449b-5p (bottom) was determined by qRT-PCR (*n* = 3, with the control expression set to 1). The statistical significance (if any) of each pairwise comparison to the vehicle control is indicated with * for *p* < 0.05. Error bar, s.e.m.

In our miRNA-seq analysis, miR-449a and miR-449b-5p were significantly upregulated in ovarian samples from HR women treated with flutamide. Here, we first examined whether this relationship holds in experimental cell models before performing further mechanistic tests. To this end, we examined two ovarian cancer cell lines, SKOV3 and Hey, alongside primary ovarian epithelial cells. In all three ovarian cell models tested, we observed that both miR-449a and miR-449b-5p were upregulated upon flutamide treatment, as measured by qRT-PCR (Fig. 3). This upregulation was statistically significant (*p* < 0.05) in the two ovarian cancer cell lines (SKOV3 and Hey). In primary ovarian epithelial cells, the upregulation of miR-449a and miR-449b-5p following flutamide treatment was less pronounced and statistically non-significant (Fig. 3). The observed difference in flutamide-induced miR-449a and miR-449b-5p expression between ovarian cancer cells and primary cells could be attributed to multiple factors, which are discussed in detail in the Discussion section.

*miR-449a and miR-449b-5p suppress CSF1R expression at both the mRNA and protein levels in ovarian cells*.

Next, we examined whether miR-449a and miR-449b-5p inhibit CSF1R expression as predicted computationally. To this end, we introduced the mimics of these miRNAs into each of the three ovarian cell models (Hey, SKOV3, and primary) and measured the corresponding CSF1R mRNA and protein levels. First, in Hey cells, we observed that mimics of miR-449a and miR-449b-5p, but not miR-449c-5p, showed pronounced effects in reducing the CSF1R expression at both the mRNA and protein levels compared to the mock control (Suppl. Figure 5). These results are consistent with the computational prediction that miR-449a and miR-449b-5p, but not miR-449c-5p, target and suppress CSF1R (Suppl. Figure 3). We therefore continued to focus on testing miR-449a and miR-449b-5p, not miR-449c-5p, in subsequent tests.

**Fig. 4:**
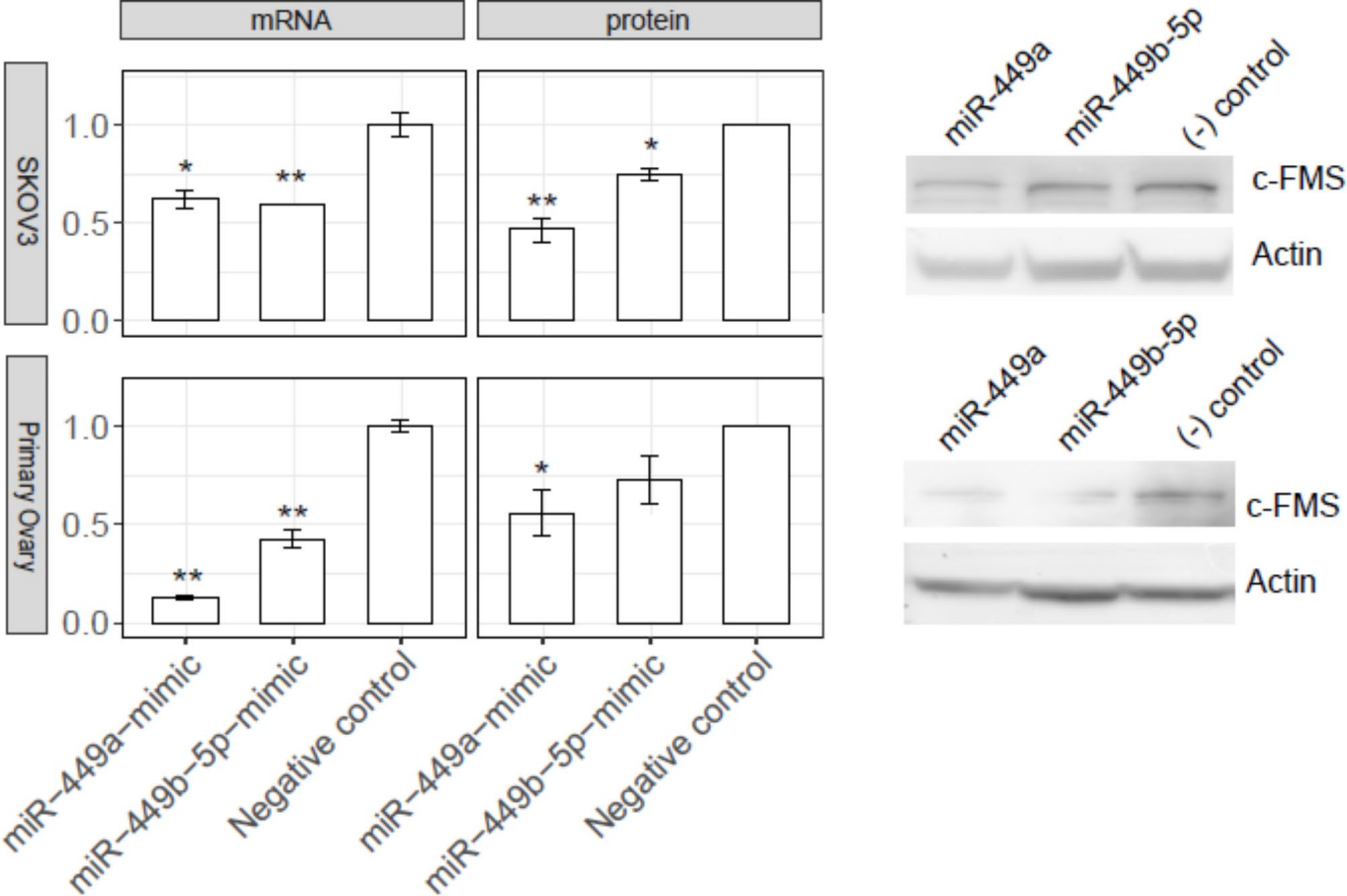
miR-449a and miR-449b-5p suppress CSF1R expression at both the mRNA and protein levels in ovarian cells. Upper panel, SKOV3 cells; Lower panel, Primary ovary cells. The mRNA level was determined by qRT-PCR (*n* = 3) and the protein level by Western blot (*n* = 2, with a representative blot shown to the right and the full original blots shown in Suppl. Figure 6). The statistical significance (if any) of each pairwise comparison to the negative control is indicated with * and ** for *p* < 0.05 and 0.01, respectively. Error bar, s.e.m.

Similar results as in Hey cells were observed in SKOV3 ovarian cancer cells (top, Fig. 4) and the primary ovarian epithelial cells (bottom, Fig. 4). In both cell models, the introduction of miR-449a mimic led to a significant decrease in CSF1R mRNA and protein levels compared to the mock controls; the introduction of miR-449b-5p mimic resulted in a similar decrease in CSF1R mRNA and protein levels as with the miR-449a mimic, although the effect appeared less pronounced (and that on CSF1R protein in primary ovarian epithelial cells was insignificant), as seen in Fig. 4.

*miR-449a and miR-449b-5p repress AR expression at both the mRNA and protein levels in ovarian cancer cells*.

**Fig. 5:**
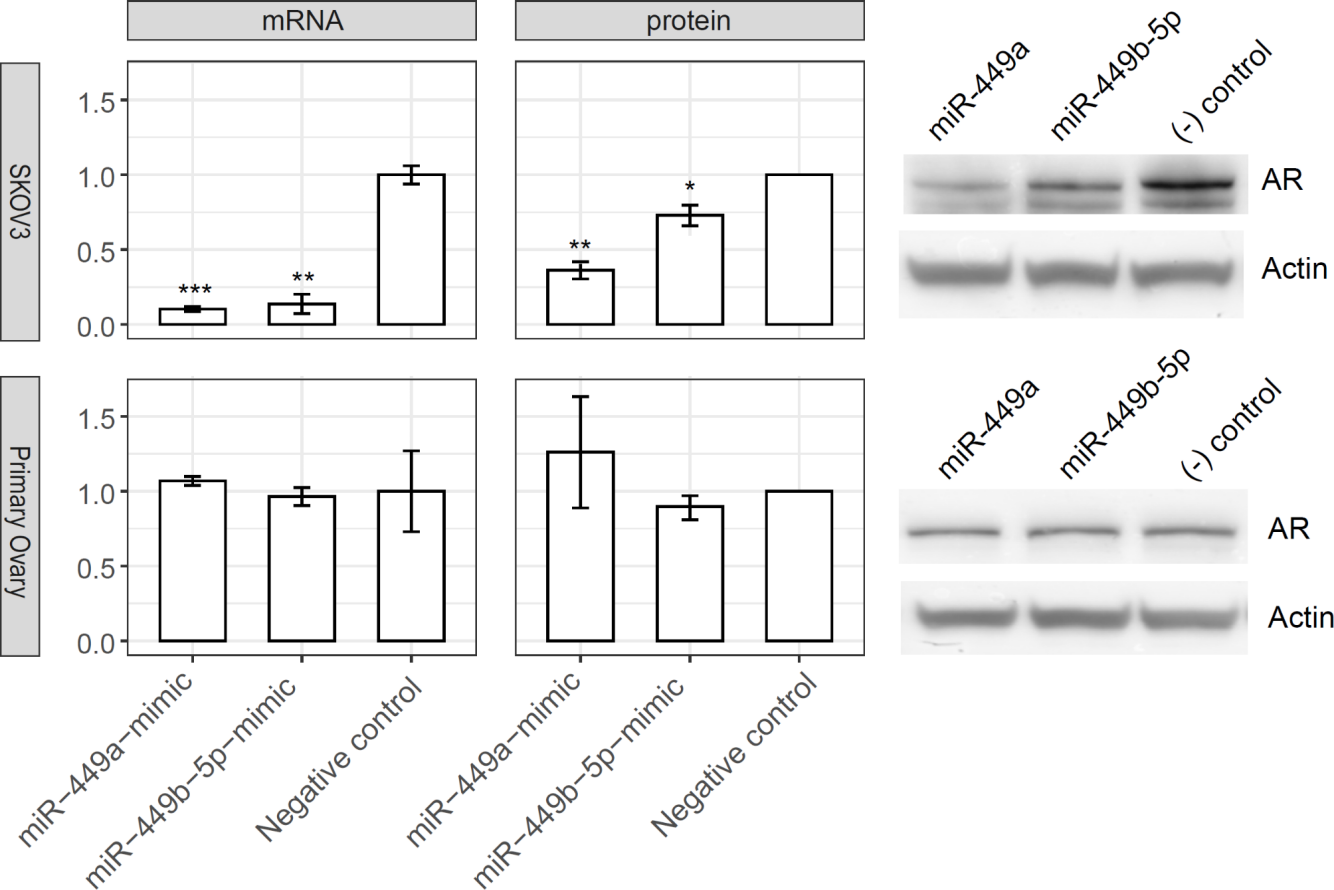
miR-449a and miR-449b-5p repress AR expression at both the mRNA and protein levels in ovarian cells. Upper panel, SKOV3 cells; Lower panel, Primary ovary cells. The mRNA level was determined by qRT-PCR (*n* = 3) and the protein level by Western blot (*n* = 2, with a representative blot shown on the right and the full original blots shown in Suppl. Figure 8). The statistical significance (if any) of each pairwise comparison to the negative control is indicated with *, **, and *** for *p* < 0.05, 0.01, and 0.001, respectively. Error bar, s.e.m.

We subsequently examined whether miR-449a and miR-449b-5p inhibit AR expression as predicted computationally. To this end, we introduced the mimics of miR-449a and miR-449b-5p into the three ovarian cell lines (SKOV3, Hey, and Primary) and measured the corresponding AR mRNA and protein levels. We observed that miR-449a and miR-449b-5p mimics significantly reduced the AR mRNA and, consistently, AR protein levels, compared to the mock control in both ovarian cancer cell lines tested (SKOV3, upper panel, Fig. 5; Hey, Suppl. Figure 7).

We did not observe a reduction in AR mRNA or protein levels following the introduction of miR-449a and miR-449b-5p mimics in primary ovarian epithelial cells (lower panel, Fig. 5). This discrepancy could be two-fold. *I*) The baseline levels of miR-449a and miR-449b-5p are significantly higher in primary ovarian epithelial cells than in Hey and SKOV3 ovarian cancer cells (Suppl. Figure 4). Consequentially, the relative increase of these miRNAs via the introduced mimics is smaller in primary ovarian cells than in Hey and SKOV3 cells, likely leading to a less pronounced effect. *II*) The target scores of miR-449a and miR-449b-5p against AR (= 56 for both miRNAs) are lower than against CSF1R (= 73 and 68, respectively; Suppl. Figure 3), indicating weaker interactions of these miRNAs with AR than with CSF1R mRNA. Consequently, the repressive effects on AR in primary ovarian cells are expected to be mild, considering both the small fold increase of miR-449a and miR-449b-5p levels via the introduced mimics and their potentially lower targeting efficiency to AR. This contrasts with the stronger effects of these miRNAs either on targeting AR in SKOV3 (Fig. 5, top) and Hey (Suppl. Figure 7) cells, or on targeting CSF1R (Fig. 4, bottom) versus AR in primary cells, in line with our experimental results.

*miR-449a and miR-449b-5p inhibit the directed migration of ovarian cancer cells*

**Fig. 6:**
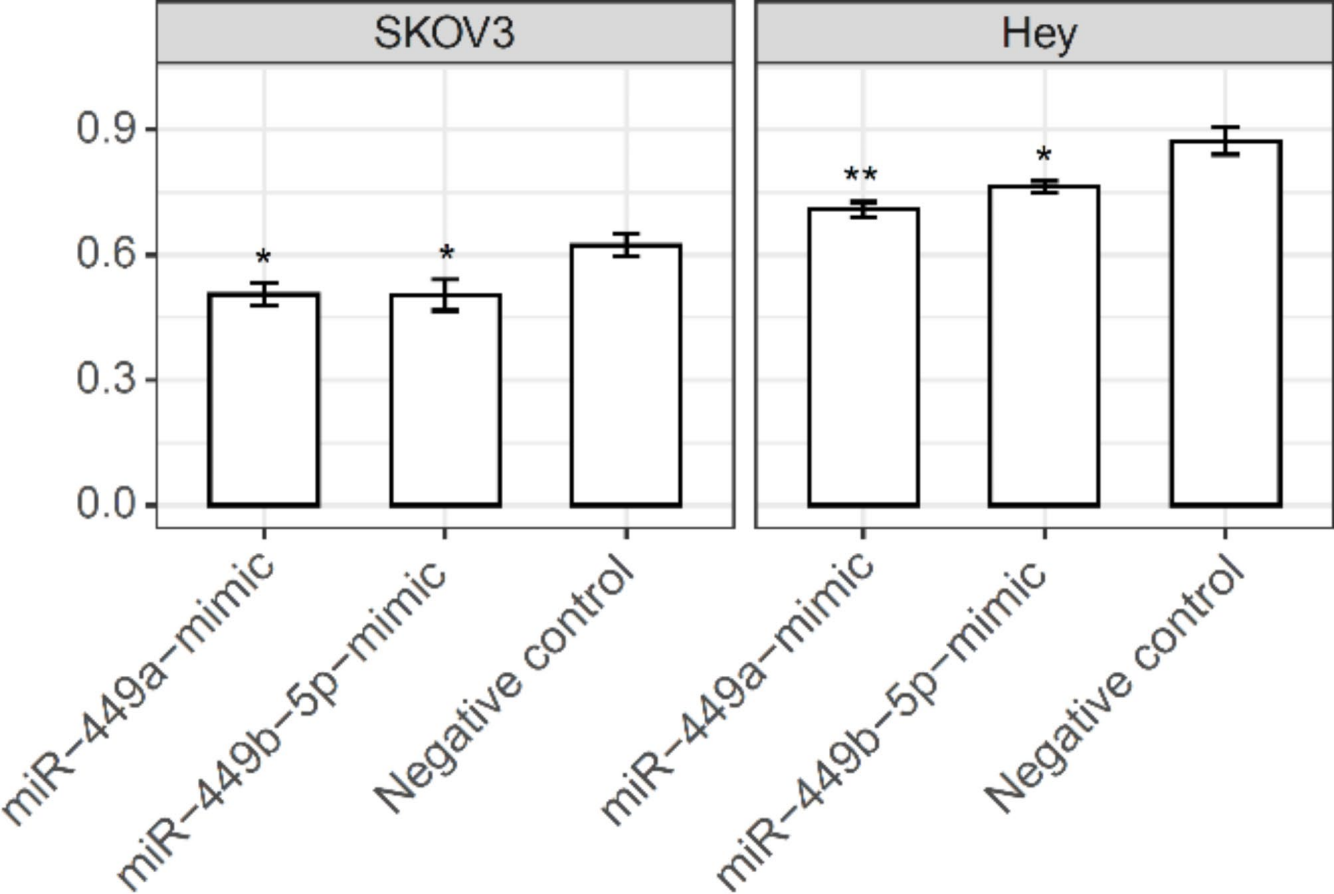
miR-449a and miR-449b-5p mimics decrease the directed motility of SKOV3 and Hey cells. Directed motility using fibronectin was performed as described in Methods (*n* = 3). Y-axis, the ratio of migrated cell number / seeded cell number as measured by a fluorimetric assay (see Methods). The statistical significance (if any) of each pairwise comparison to the negative control is indicated with * and ** for *p* < 0.05 and 0.01, respectively. Error bar, s.e.m.

Since miR-449a and miR-449b-5p suppress CSF1R and AR expression in ovarian cancer cells (SKOV3 and Hey) and CSF1R and AR are associated with the risk of ovarian cancer development, we next tested whether these miRNAs could exert any noticeable effects on the behavior of ovarian cancer cells. To this end, we introduced miR-449a and miR-449b-5p mimics into SKOV3 and Hey cells. The endogenous baseline levels of these miRNAs are lower in these ovarian cancer cells than in primary ovarian epithelial cells (Suppl. Figure 4), and thus, the effects of the introduced mimics may be better observed. We focused on the study of directed cell migration because our previous work had shown that DHT (dihydrotestosterone, a potent androgen) increased the motility of tumorigenic ovarian epithelial cells^9^. We measured directed cell migration in SKOV3 and Hey cells transfected with miR-449a and miR-449b-5p mimics versus the mock control. As seen in Fig. 6, both miR-449a and miR-449b-5p mimics induced a moderate, yet statistically significant, reduction in cell migration in both SKOV3 and Hey cells.

## Discussion

Dysregulated miRNAs, such as the miRNA-200 and let-7 families have been implicated in ovarian cancer diagnosis and progression^25,26^. However, prior publications have focused on tissues from women already diagnosed with the disease. To our knowledge, our study is the first to investigate miRNA dysregulation in normal tissues from women at HR for ovarian cancer, making our findings unique in this regard.

A notable outcome of transcriptomic miRNA profiling of ovarian and fallopian tube tissues from women at HR for ovarian cancer was the general trend of miRNA upregulation in response to flutamide, similar to that observed in LR versus HR tissues. This analysis, as well as our prior work^9,10^, is consistent with the role of flutamide in normalizing the expression of dysregulated gene markers in HR tissues.

For our in vitro validations, we focused on miR-449a and miR-449b-5p, which are among the most significantly up-regulated miRNAs (> 4-fold, Fig. 1A) in response to flutamide treatment and computationally predicted to target and inhibit the expression of CSF1R and AR. The tumor-suppressive roles of miR-449a and miR-449b-5p are known in various cancers, including gastric, colon, prostate, and breast cancers^27–29^. However, their role in epithelial ovarian cancer, especially high-grade serous carcinomas, has been largely unexplored^30^. Our present study bridges this knowledge gap by showing how these miRNAs modulate the expression of CSF1R and AR in ovarian cells, implicating their significant roles in regulating CSF1/CSF1R and AR signaling pathways during the development of epithelial ovarian cancer in HR women.

In ovarian cancer SKOV3 and Hey cells, we found that (a) flutamide significantly upregulated the expression of miR-449a and miR-449b-5p, (b) introducing mimics of miR-449a and miR-449b-5p downregulated CSF1R and AR expression at both mRNA and protein levels, and (c) miR-449a and miR-449b-5p exhibited inhibitory effects on cell migration (Figs. 3, 4, 5 and 6; Suppl. Figures 5 & 7). Collectively, these experimental data validated the links *a*,* b*, and *c* proposed in our working model (Fig. 2) and demonstrated the effect of flutamide, via the expression of miR-449a and miR-449b-5p, on the suppression of CSF1R and AR and cancer cell migration.

We found that miR-449a and miR-449b-5p were significantly less expressed in SKOV3 and Hey ovarian cancer cell lines compared to the primary ovarian cell control (Suppl. Figure 4), which aligns with their lower expression pattern in HR subjects related to LR patients (Fig. 1B). Although as noted above, HR samples were morphologically and pathologically normal and cancer-free, these observations suggest a consistent trend of miR-449a and miR-449b-5p downregulation across the spectrum of ovarian cancer risk and progression.

The lower baseline levels of these miRNAs in ovarian cancer cells compared to primary cells may allow for a more pronounced relative increase upon flutamide treatment, consistent with the observation in Fig. 3. However, this difference may also be due to other mechanisms. First, it is known that the expression of miR-449a and miR-449b-5p can be upregulated by E2F1^31,32^, by autophagy via FoxO1^33^, and by activating its host gene CDC20B^34,35^. These pathways may be more responsive to flutamide treatment in ovarian cancer cells than in primary cells, through mechanisms dependent on or independent of flutamide’s antiandrogen effect on the AR pathway. Second, miR-449a and miR-449b-5p are reported to be epigenetically silenced in cancers via DNA methylation and histone modifications^32,36^. Flutamide treatment may reverse this silencing, leading to a more pronounced upregulation of these miRNAs in cancer cells compared to primary cells. The exact mechanisms remain to be elucidated in future studies.

Interestingly, the miR-449 family is part of the broader miR-34/miR-449 superfamily, which comprises two miRNA clusters, miR-34a/b/c and miR-449a/b/c. These clusters share a similar seed sequence and adjacent nucleotide sequences^37^and overlapping functions in mediating cell cycle arrest and apoptosis in tumor suppression^35^. Previous studies have linked the downregulation of miR-34 with ovarian cancer^38,39^. In this study, we observed that some members of the miR-34 family followed a similar expression trend to miR-449 in ovarian tissues, with miR-34b-5p expression significantly upregulated upon flutamide treatment (Suppl. Figure 1 A, left) and miR-34c-5p/3p expression significantly upregulated in LR compared to HR subjects (Suppl. Figure 1B, left). This trend of miR-34 expression was absent in fallopian tube samples (Suppl. Figure 1, right), consistent with the more subdued differential expression of miR-449 in the fallopian tube compared to the ovary (Fig. 1B).

The involvement of the CSF1/CSF1R pathway in the progression of epithelial ovarian cancer is well-documented^40^. Recent findings support the blockade of CSF1R in vivo in cancers leading to an immune permissive tumor microenvironment^41^. Regarding the potential role of CSF1/CSF1R as functional biomarkers of ovarian cancer initiation, our mouse models previously demonstrated the enhanced virulence associated with CSF1 overexpression in ovarian cancer and, conversely, reduced tumorigenicity in mice implanted with CSF1-negative ovarian cancer cells^42^. These results, combined with our previous observations of CSF1 and CSF1R overexpression in normal HR human tissues when compared to LR tissues^9,10^, suggested the critical role of the CSF1/CSF1R pathway in ovarian cancer initiation. Given this, our current finding that flutamide can restore the reduced expression of miR-449a and miR-449b-5p in HR tissues, thereby suppressing CSF1R, provides a mechanistic insight into how flutamide may potentially mitigate the risk of ovarian cancer initiation.

The putative role of the AR pathway in ovarian cancer development was a primary motivator for our Phase 2 clinical study, in which we tested the effects of the anti-androgen agent flutamide in HR women^9^. Androgen-treated ovarian epithelial cells show increased proliferation and decreased cell death^43^. Androgen dysregulation was found in BRCA mutation-derived ovarian epithelial cells relative to control cells, which is significant as BRCA mutations account for the majority of hereditary ovarian cancer risk, and such altered responses may be involved in ovarian carcinogenesis^44^. In addition, a study of androgen ablation in male nude mice implanted with p53-mutated ovarian serous cells demonstrated 24-fold less evidence of ovarian cancer development when compared with intact male nude mice^9^. Androgen-related ovarian carcinogenesis has been reviewed^45^. Our findings that flutamide effectively normalized the elevated protein biomarkers in HR patients lend further support to the importance of androgen signaling and the potential role of AR as a functional biomarker in the initiation and progression of ovarian cancer. The present study extends these findings by demonstrating that flutamide upregulates miR-449a and miR-449b-5p in SKOV3 and Hey ovarian cancer cells (Fig. 3), leading to a marked reduction of AR expression at both the mRNA and protein levels (Fig. 5 and Suppl. Figure 5). This revelation of miR-449-mediated suppression of AR expression (confirmed link *c* in our working model, Fig. 2) adds a new dimension to our understanding of flutamide action, complementing its established direct antagonistic binding to AR. That is, effectively, flutamide emerges as a ‘dual inhibitor’ of AR signaling pathway.

Our study is unique in its focus on investigating miRNAs in normal ovarian and fallopian tube tissues from women at high risk for ovarian cancer but without existing ovarian or other peritoneal cancers, tubal dysplasia, or STIC lesions. There is a paucity of data on miRNA profiling of HR/LR tissues in the current literature, and there are no reports of miR449 dysregulation in HR/LR tissues. Our findings suggest that flutamide treatment can effectively restore the diminished expression of miR-449a and miR-449b-5p, among other miRNAs, in the ovarian and tubal tissues of these high-risk women. This restoration decreases the expression of critical functional molecular markers such as CSF1R and AR, which are potentially associated with an elevated risk for ovarian cancer.

Our study has several limitations. First, we employed a uniform sub-toxic dose (5 µM) of flutamide^9^ to examine its effect on miR-449 expression across tested cell lines (Fig. 3). Future studies examining the inhibition of cell viability or proliferation by flutamide (IC50 values) would further characterize its potential therapeutic effects. Second, while we demonstrated the effects of miR-449a and miR-449b-5p on CSF1R and AR expression in vitro, the complex in vivo interactions and long-term effects remain to be elucidated. Lastly, our patient cohort for the miRNA-seq analysis, although carefully selected, was relatively small, necessitating larger studies to validate and expand upon our findings on flutamide-induced miRNA expression profile changes.

Despite these limitations, our research supports the potential of a well-tolerated low-dose flutamide regimen as a chemopreventive measure for ovarian cancer^9^. This is particularly promising for high-risk women with decreased miR-449 expression, who may represent the subgroup most likely to benefit. Moving forward, further studies are needed to ascertain whether the flutamide-induced miR-449 restoration effectively translates into a reduced risk of developing ovarian cancer in these patients.

## Electronic supplementary material

Below is the link to the electronic supplementary material.

Supplementary Material 1

## Data Availability

The miRNA-sequencing reads have been deposited and are available in the Gene Expression Omnibus (GEO) database (GSE252170).

## Abbreviations

AR: Androgen Receptor
BMI: Body Mass Index
BRCA1/2: Breast Cancer 1/2, early onset (genes)
CSF1: Colony-Stimulating Factor 1
CSF1R: Colony-Stimulating Factor 1 Receptor
FFPE: Formalin-Fixed, Paraffin-Embedded
GAPDH: Glyceraldehyde-3-Phosphate Dehydrogenase
hEGF: human Epidermal Growth Factor
HR: High-Risk
IIT: Investigator-Initiated Trials
LR: Low-Risk
miR: microRNA
qRT-PCR: Quantitative Reverse Transcription Polymerase Chain Reaction
STIC: Serous Tubal Intraepithelial Carcinoma
