# Supplementary material for: miR-449, identified through antiandrogen exposure, mitigates functional biomarkers associated with ovarian cancer risk
Source: Sci Rep. 2024 Dec 2;14:29937. doi: 10.1038/s41598-024-80173-z (PMC11611913; doi:10.1038/s41598-024-80173-z)
Supplement: Supplementary file 1 — Supplementary Material 1 [file 41598_2024_80173_MOESM1_ESM.pdf]

A) Flutamide vs. HR (left, ovary; right, tube)

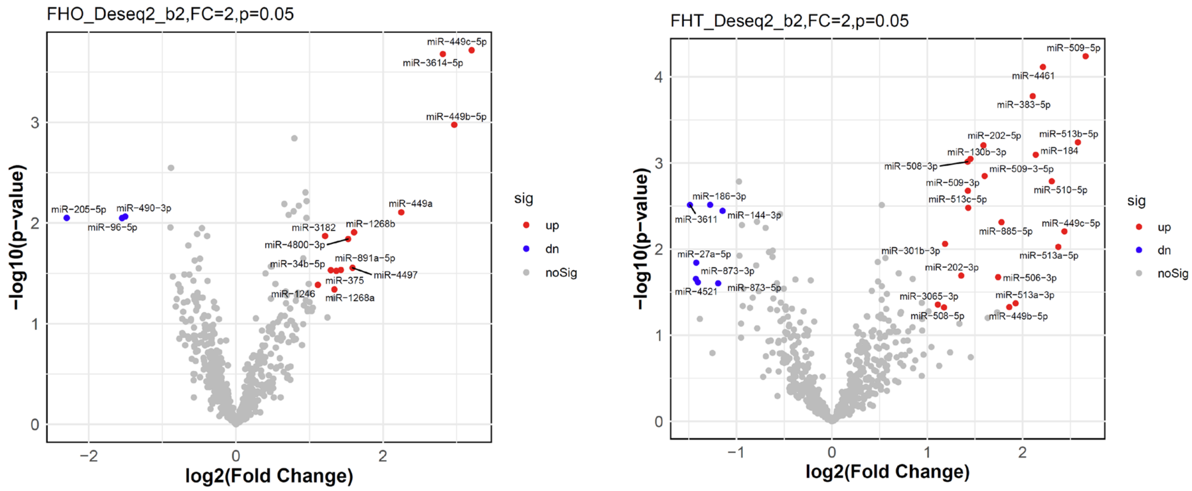

B) LR vs. HR (left, ovary; right, tube)

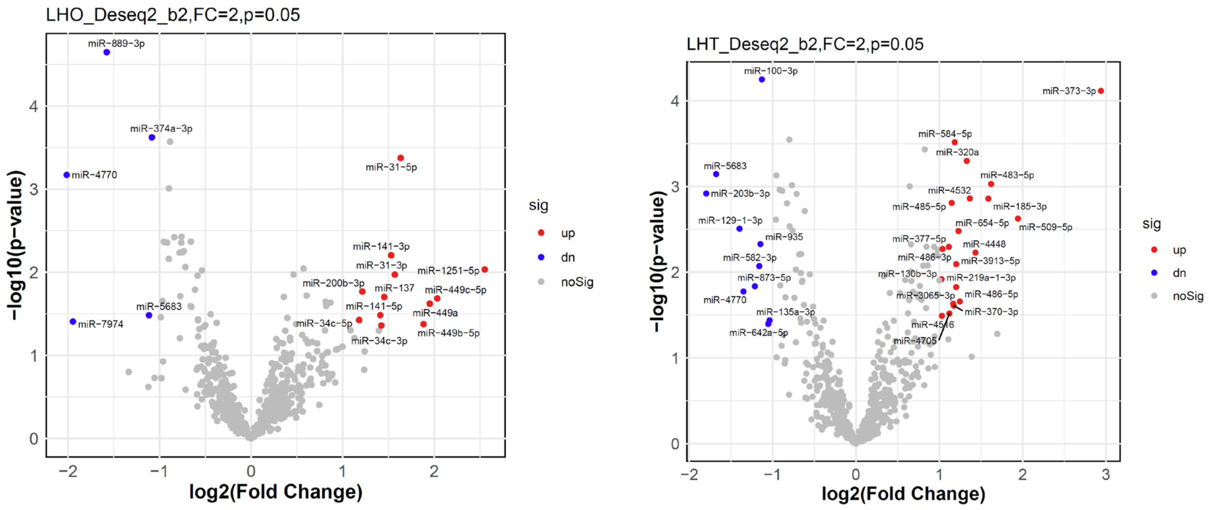

Suppl. Figure 1. Volcano plots of miRNA expression profiles in flutamide vs. HR cohort (A) and in LR vs. HR cohort (B) from the ovary (left) and fallopian tube (right) samples. Red and blue dots indicate miRNAs that are significantly up-regulated (fold change > 2, $p < 0.05$) and down-regulated (fold change < 0.5, $p < 0.05$), respectively. The statistical significance (p-value) of the number difference between red and blue dots is 0.018 (left, A), 0.004 (right, A), 0.15 (left, B), and 0.05 (right, B), respectively.

A)

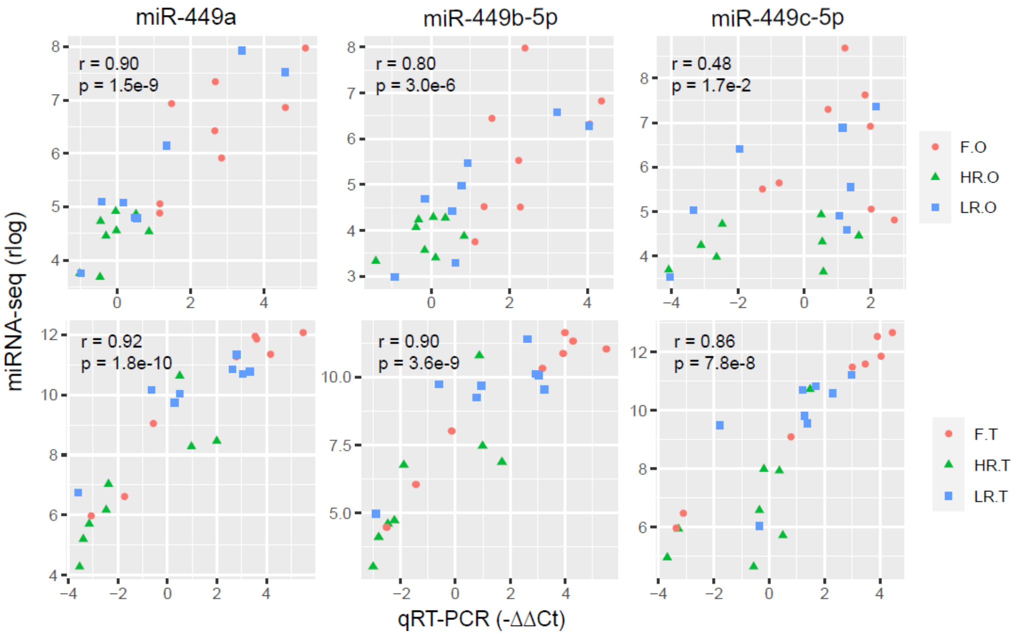

B)

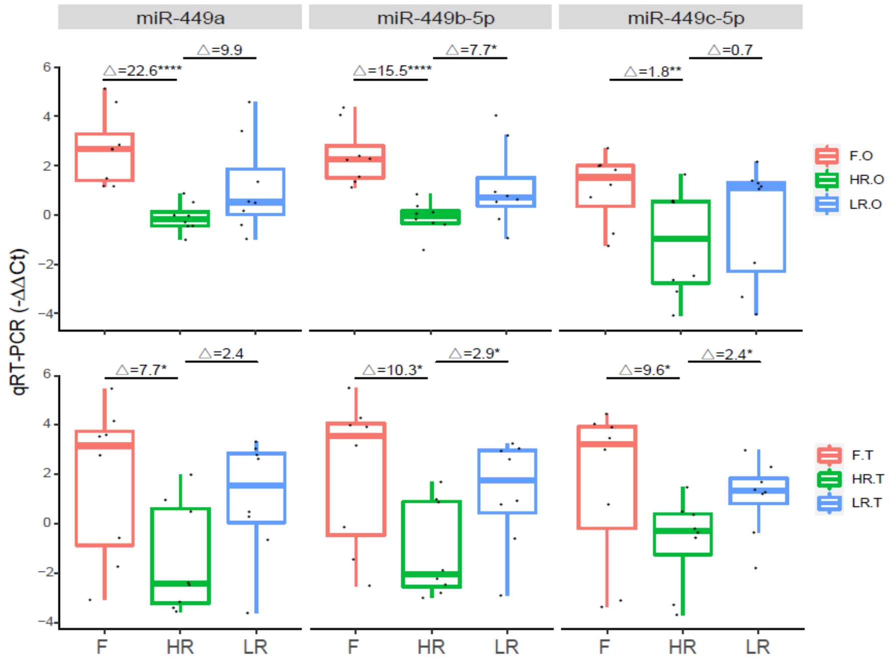

Suppl. Figure 2. qRT-PCR Validation of miRNA-seq Results. In each patient cohort (Flutamide/F, HR, and LR) from the miRNA-seq analysis, qRT-PCR was used to assess miR-449 expression in ovarian and fallopian tube samples from eight randomly selected individuals. (A) Correlation analysis for miR-449a (left), miR-449b-5p (middle), and miR-449c-5p (right) in ovarian (top) and tube (bottom) samples, between miRNA-seq results (y-axis, rlog normalized values) and qRT-PCR (x-axis, log2 of miRNA expression relative to the HR average). Each panel shows the Pearson correlation coefficient (r) and the p-value of the correlation. (B) Boxplot of miR-449 expression levels determined by qRT-PCR (y-axis, log2 of miRNA expression relative to the HR average) in ovarian (top) and tube (bottom) samples. Glass's delta effect size and p-values (*, **, and **** indicating $p < 0.05$, 0.01, and 0.0001, respectively) for each pairwise comparison are shown at the top of each boxplot.

miR-449a:

**miRNA Name**	hsa-miR-449a	**miRNA Sequence**	UGGCAGUGUAUUGUUAGCUGGU
**Previous Name**	hsa-miR-449		
**Target Score**	73	**Seed Location**	192
**NCBI Gene ID**	1436	**GenBank Accession**	NM_005211
**Gene Symbol**	CSF1R	**3' UTR Length**	795
**Gene Description**	colony stimulating factor 1 receptor		

**miRNA Name**	hsa-miR-449a	**miRNA Sequence**	UGGCAGUGUAUUGUUAGCUGGU
**Previous Name**	hsa-miR-449		
**Target Score**	59	**Seed Location**	1166
**NCBI Gene ID**	367	**GenBank Accession**	NM_001011645
**Gene Symbol**	AR	**3' UTR Length**	6783
**Gene Description**	androgen receptor		

miR-449b-5p:

**miRNA Name**	hsa-miR-449b-5p	**miRNA Sequence**	AGGCAGUGUAUUGUUAGCUGGC
**Previous Name**	hsa-miR-449b		
**Target Score**	68	**Seed Location**	192
**NCBI Gene ID**	1436	**GenBank Accession**	NM_005211
**Gene Symbol**	CSF1R	**3' UTR Length**	795
**Gene Description**	colony stimulating factor 1 receptor		

**miRNA Name**	hsa-miR-449b-5p	**miRNA Sequence**	AGGCAGUGUAUUGUUAGCUGGC
**Previous Name**	hsa-miR-449b		
**Target Score**	59	**Seed Location**	1166
**NCBI Gene ID**	367	**GenBank Accession**	NM_001011645
**Gene Symbol**	AR	**3' UTR Length**	6783
**Gene Description**	androgen receptor		

Suppl. Figure 3. Computational prediction of miR-449a and miR-449b-5p targeting CSF1R and AR. The predictions were obtained from miRDB, an online database for miRNA target predictions and functional annotations [21].

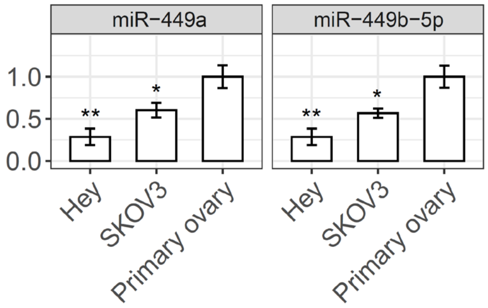

Suppl. Figure 4. The expression levels of miR-449a and miR-449b-5p in Hey and SKOV3 ovarian cancer cells and primary ovarian epithelial cells, measured by qRT-PCR. The statistical significance (if any) of each pairwise comparison to the primary ovarian cell control is indicated with * and ** for p < 0.05 and 0.01, respectively. Error bar, s.e.m.

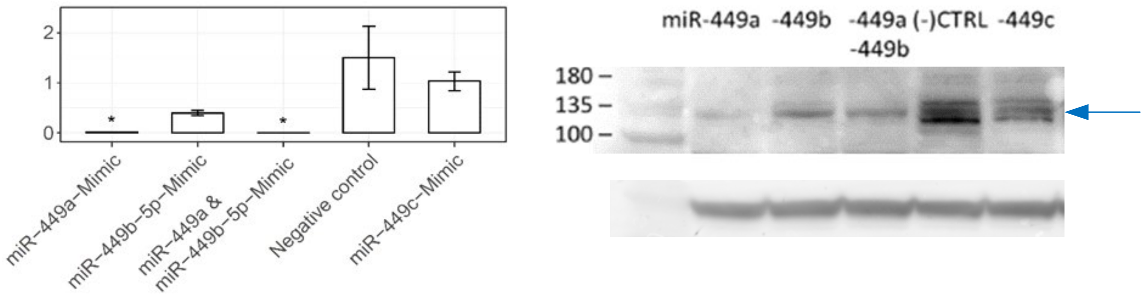

Suppl. Figure 5. CSF1R mRNA and protein in Hey cells with miR-449 mimics. (Left) CSF1R mRNA levels at indicated conditions, measured by qRT-PCR (n=3). The statistical significance (if any) of each pairwise comparison to the negative control is indicated with * for p < 0.05. Error bar, s.e.m. (Right) CSF1R protein levels at indicated conditions measured by Western blot (with the full original blots shown in Suppl. Fig. 9). Dual mimics (miR-449a and miR-449b-5p) showed a similar effect as a single miR-449a mimic on CSF1R mRNA and an intermediate effect between miR-449a and miR-449b-5p on CSF1R protein. miR-449c-5p had little effect on CSF1R mRNA and protein.

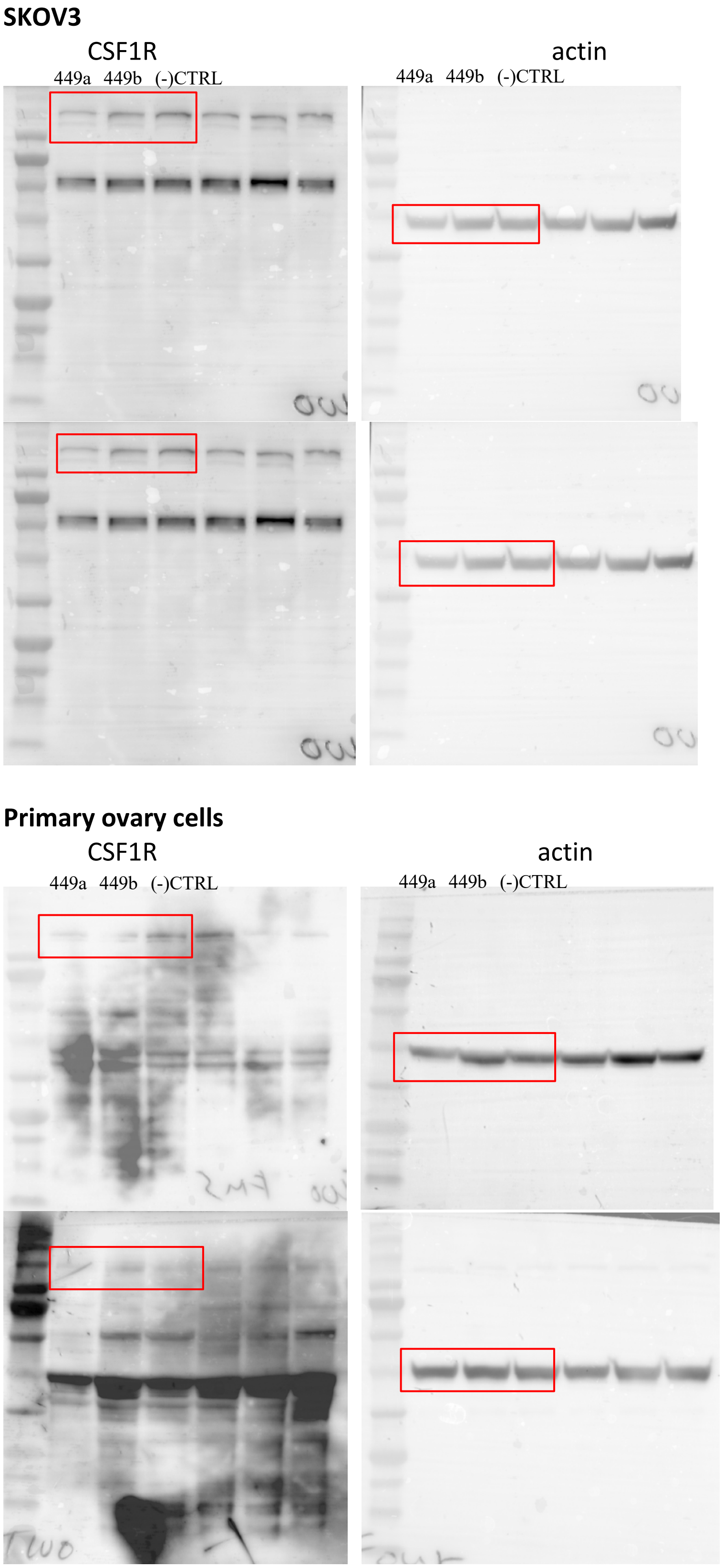

Suppl. Figure 6. Full original blots of the Western results in Fig. 4.

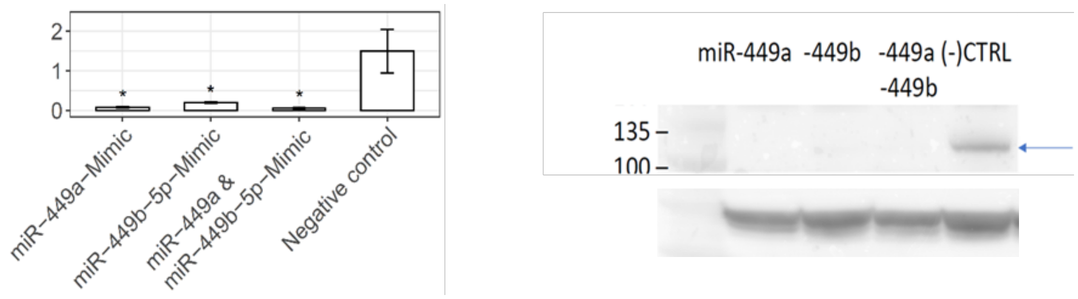

Suppl. Figure 7. AR mRNA and protein in Hey cells with miR-449a and miR-449b-5p mimics. (Left) AR mRNA levels at indicated conditions, measured by qRT-PCR (n=3) The statistical significance of each pairwise comparison to the negative control is indicated with * for $p < 0.05$. Error bar, s.e.m. (Right) AR protein levels at indicated conditions, measured by Western blot (with the full original blots shown in Suppl. Fig. 10).

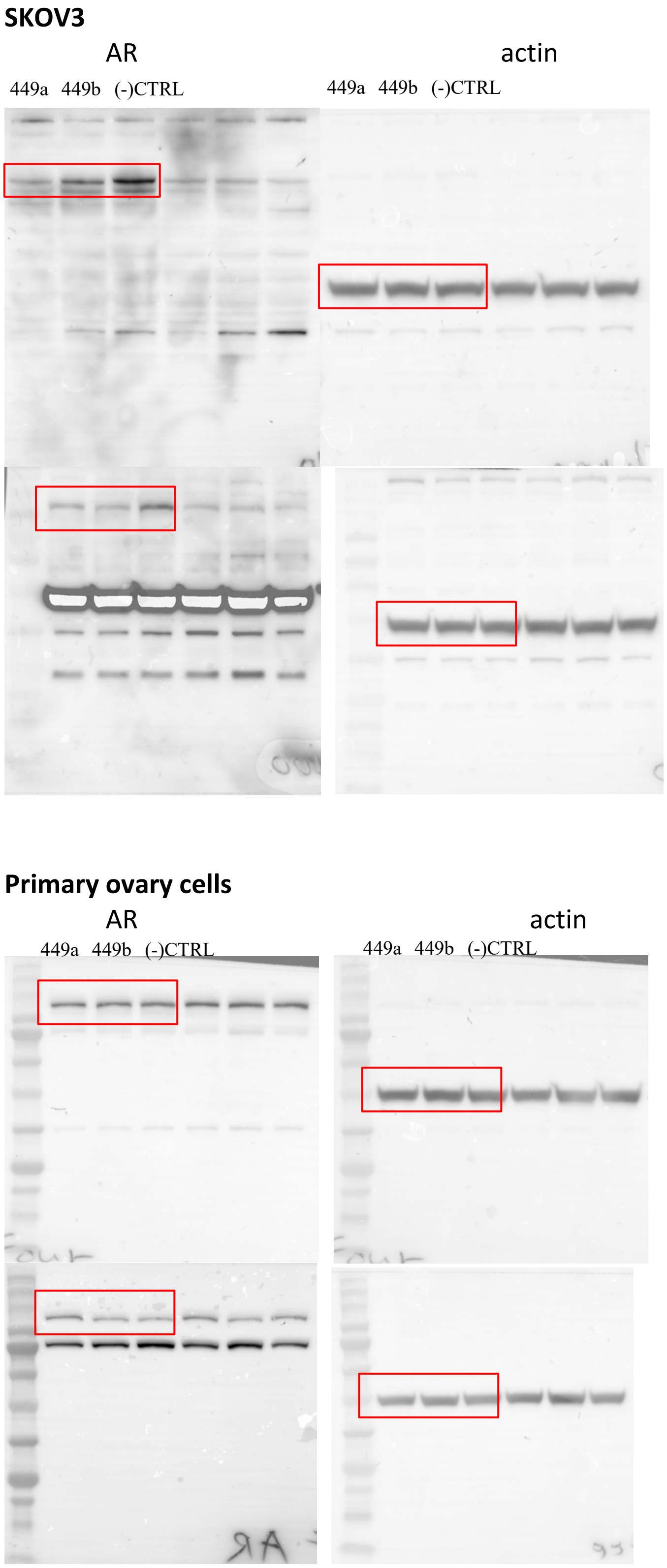

Suppl. Figure 8. Full original blots of the Western results in Fig. 5.

Hey cells

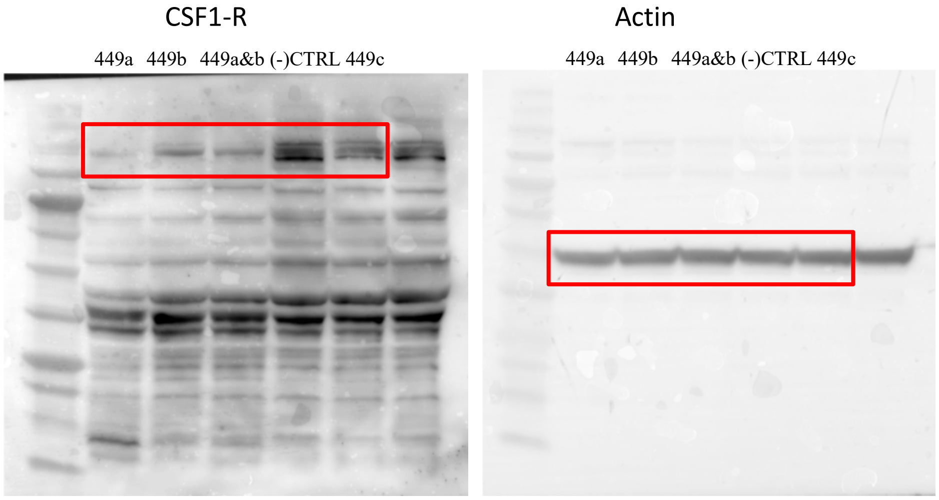

Suppl. Figure 9. Full original blots of the Western results in Suppl. Figure 5.

Hey cells

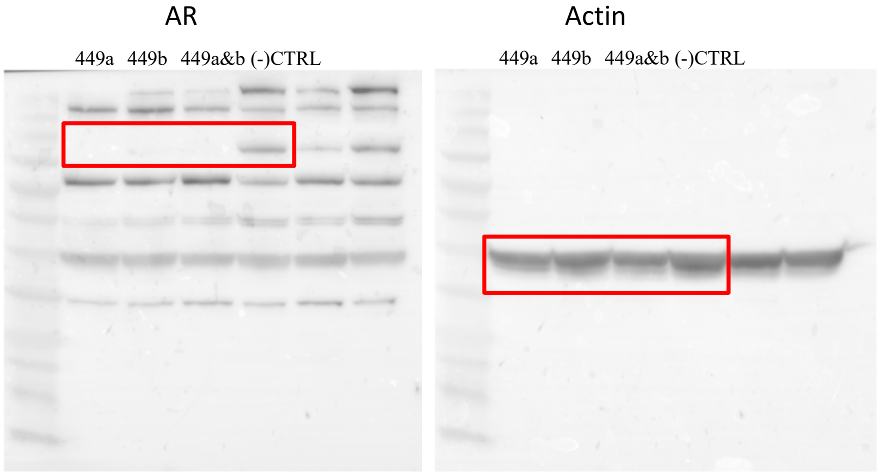

Suppl. Figure 10. Full original blots of the Western results in Suppl. Figure 7.
